# Paediatric Cushing’s disease: long-term outcome and predictors of recurrence

**DOI:** 10.3389/fendo.2024.1345174

**Published:** 2024-01-22

**Authors:** Martin O. Savage, Rosario Ferrigno

**Affiliations:** ^1^ Centre for Endocrinology, William Harvey Research Institute, Barts and the London School for Medicine & Dentistry, Queen Mary, University of London, London, United Kingdom; ^2^ UOSD di Auxologia e Endocrinologia, AORN Santobono-Pausilipon, Napoli, Italy

**Keywords:** Cushing’s, pituitary adenoma, transsphenoidal surgery, pituitary radiotherapy, recurrence

## Abstract

Paediatric Cushing’s disease (CD) is characterized by excess ACTH secretion from a pituitary adenoma, leading to hypercortisolism. It has approximately 5% of the incidence of adult CD and is a rare disorder in the paediatric age range. The four most specific presenting features of hypercortisolism are: change in facial appearance, weight gain, decreased linear growth and virilisation shown by advanced pubic hair for the stage of breast development or testicular volume. The main diagnostic priority is the demonstration of hypercortisolism followed by distinction between its ACTH-dependent and ACTH-independent origin, thus leading to identification of aetiology. All treatment options aim to resolve or control hypercortisolism. Consensus favours transsphenoidal (TSS) pituitary surgery with selective removal of the corticotroph adenoma. TSS in children with CD is now well established and induces remission in 70-100% of cases. External pituitary radiotherapy and bilateral adrenalectomy are second-line therapeutic approaches in subjects not responding to TSS. Long-term medical treatment is less frequently adopted. Recurrence in paediatric CD cases is low with factors predicting relapse being higher post-TSS cortisol and ACTH levels and rapid recovery of the hypothalamic-pituitary-adrenal axis after TSS. In summary, complete excision of the microadenoma with histological and biochemical evidence for this, predicts a low rate of recurrence of CD. Due to the need for rapid diagnosis and management to avoid the burden of prolonged exposure to hypercortisolism, tertiary university centres comprising both paediatric and adult endocrinology specialists together with experienced pituitary surgery and, eventually, radiotherapy units are recommended for referral of these patients.

## Introduction

Cushing’s disease (CD), characterised by hypercortisolism due to excess ACTH secretion by a pituitary adenoma, is essentially an adult disorder which occasionally presents in children (1, 2). Its incidence in the paediatric age range is considered to be approximately 5% of that seen in adults (3). Consequently, a paediatric endocrinologist is likely to see only a few cases during their career, which strengthens the case for specialist university centres, staffed by both paediatric and adult endocrinologists, neurosurgeons specialising in transsphenoidal pituitary surgery, and specialised pituitary radiotherapists, to be the optimal institutions for the care of these patients. There is no doubt that collaboration and cooperation between paediatric and adult endocrinologists in the components of clinical management is beneficial to patient care (4).

Because the morbidity of hypercortisolism, also named Cushing’s syndrome (CS), in children is serious, early and rapid investigation is indicated in suspected cases. Two main goals dominate the investigational process. First, it is essential to confirm or exclude the presence of endogenous CS. Secondly, the etiologic cause of CS needs to be defined. Cushing’s syndrome can be divided into two main aetiological groups, namely ACTH-dependent and ACTH-independent CS: the first group comprises CD and ectopic ACTH syndrome, whereas the second one comprises adrenal cortical neoplasms, both benign and malignant, and adrenocortical hyperplasia. Exogenous glucocorticoid administration should be ruled out in the early phases of the diagnostic work-up to avoid needless investigations.

## Clinical presentation of paediatric CD

There are four key presenting features which are currently recognised to have a relevant diagnostic value (**Table 1**), whereas other features tend to be non-specific and are therefore less reliable in suspecting CD in children. These four key diagnostic features are; change in facial appearance with rounding of the face; weight gain, which is almost universal; retardation of linear growth, which may or may not lead to clinical short stature (i.e. height <-2 SDS); and increased virilisation (4). Although being present in 100% of cases in an English series of 41 patients (7), the change in facial appearance usually occurs slowly over several months or years, thus it may not be recognised as being pathological by parents or family doctors. In the same English series, weight gain was present in 98% of cases, and it usually leads to a marked increase in few months, thus being more easily recognized as a pathological sign by patients’ relatives. Growth retardation was present in all cases where low height velocity was documented, but actual short stature was reported only in 42% (10) and 56% of cases (6) in two large paediatric CD series, so short stature should not be considered as a CS sign *per se (* 11). Virilisation presents as a disharmony of secondary sexual characteristics with Tanner stage pubic hair growth inappropriately advanced compared to breast development or testicular volume (12), although it may not be easy to identify in patients which are already in pubertal age. Other features which are less specific, but nevertheless important, include osteopaenia, hirsutism, mood changes, headache, striae, hypertension, acne, and pubertal delay (4). Bone age is usually within the normal range and not significantly decreased despite the short stature. Increased adrenal androgen secretion may contribute to this (12). Hypertension was common at diagnosis, being present in 36 -71% of cases (**Table 1**). Growth hormone responses to stimulation are usually normal at presentation, however gonadotrophin deficiency is a complication of long-term hypercorticism and the combination of decreased testicular volume or decreased breast development and advanced pubic hair growth is suggestive of Cushing’s syndrome (12).

**Table 1:** Frequency of clinical findings at diagnosis of paediatric Cushing’s disease.

	Devoe 1997 (5)	Shah 2011 (6)	Storr 2011 (7)	Lonser 2013 (8)	Guemes 2016 (9)
**Total number of patients**	42	48	41	200	16
**Mean age/median age (range)**	13.4 y^a^ (6.5-18)	14.85^b^ ± 2.5 y (9-19)	12.3 ^b^ ± 3.5 y (5.7-17.8)	10.6 ^b^ ± 3.6 y (4-19)	10 y^a^ (7-15.5)
Clinical symptoms and signs (%)					
**Weight gain**	92	98	98	93	94
**Growth retardation**	84	83	100	63^c^	63
**Short stature**		56			
**Facial changes**	46	98	100	63	
**Irregular menses (females)**				49^d^	
**Osteopaenia**	74				
**Fatigue or weakness**	67		61	48	38
**Hirsutism**	46		59	56	38
**Virilization**			76		
**Psychiatric disorders**	44^e^			31^f^	
**Mood changes**		46	59^g^		44^h^
**Headache**	26		51	38	
**Striae**	36	58	49	55	44
**Hypertension**	63	71	49	36	50
**Acne**	46		44	47	50
**Pubertal delay or arrest**	60				19
**Early secondary sexual development**					31
**Easy bruising**	28	17		25	19
**Dorsal cervical or supraclavicular fat pad**	28			69	
**Hyperpigmentation**					13
**Acanthosis nigricans**		75		32	
**Muscle weakness**		48			
**Sleep disturbances**					19
**Glucose intolerance or diabetes**		25		7	
**Bone fractures**				4	
**Hypokalaemia**					6
**Infection**		15			

Y, years; a median age; b mean age; c pre-pubertal patients (n = 91) showed growth retardation in 85%of cases, post-pubertal patients (n = 109) showed growth retardation in 44% of cases; d primary or secondary amenorrhea; e compulsive behaviour; f depression, anxiety, mood swings; g emotional lability/depression; h mental changes changes/poor school performance.

## Diagnostic investigations

Clinical skills remain important, and the history must exclude administration of exogenous glucocorticoids. Height and weight measurements, pubertal development using Tanner’s criteria and bone age assessment should also be performed. Confirmation of hypercortisolism can be assessed using three tests (4); 24-h urinary-free cortisol, late-night sleeping salivary/serum cortisol, and dexamethasone testing. None of these tests has 100% diagnostic accuracy, so multiple tests are usually required to confirm hypercortisolism. Among them, late night serum cortisol had the highest sensitivity and specificity in children (13, 14).

## Confirmation of aetiology

ACTH-dependent CS needs to be differentiated from ACTH-independent CS. First, hormonal investigations should be performed. In CD, morning plasma ACTH is typically detectable (>5 pg/ml) compared with suppressed values in primary adrenal disorders (13). The CRH stimulation test may also be useful but is not widely available. Patients with excess ACTH-secreting pituitary adenomas typically give an exaggerated response to CRH, resulting in anelevated cortisol response (15). Second, imaging techniques are required. Pituitary MRI is the optimal method for pituitary visualisation and should be performed after hormonal confirmation of ACTH-dependent CS in patients with suspect CD. However, MRI was only able to positively identify an adenoma in 16-71% of CD cases and in an English series of 41 patients appearances were consistent with a microadenoma in 55% (21/38) of paediatric patients compared with 76% (50/66) of adult patients *(P=0.045) (* 7).

Although a positive MRI is beneficial for adenoma identification and supports the diagnosis of CD, the relatively low prediction rate requires a more precise localisation technique, the bilateral inferior petrosal sinus sampling (BIPSS). BIPSS with CRH stimulation (1 µg/kg, max 100 µg/kg) has been suggested in paediatric patients with a negative MRI and confirmed hypercortisolism (16). The best practical organisation is for the BIPSS procedure to be performed by a radiologist who has extensive experience of this investigation in adults with CS. The aim of this investigation is to demonstrate excess central ACTH secretion, thereby excluding ectopic ACTH syndrome, which remains very rare in children.

## Treatment and therapeutic outcome

The overall opinion of expert adult endocrinologists treating Cushing’s disease is that the treatment of choice is removal of the microadenoma by transsphenoidal pituitary resection (TSS) (17). This neurosurgical approach was developed in the 1980s and has now become the standard therapy for Cushing’s disease. TSS can be very challenging in children because the microadenomas are often extremely small and difficult to locate and remove, as well as due to the specific features of skull base in paediatric patients, including anatomy of the sellar region varying with age, variable pneumatisation of the sphenoid bone according to age, reduced inter-carotid distance in younger children, and high frequency of anatomic variants, namely shorter nare-sellar and vomer-clivus distances and smaller transsphenoidal angles (18). Total excision of a corticotroph microadenoma results in immediate post-operative ACTH and cortisol deficiency (19). The histological appearance of normal corticotroph cells surrounding the adenoma are morphologically abnormal, the appearance being known as Crooke’s change.

## Definition of success of transsphenoidal surgery

There is no international consensus on the definition of success of TSS. The term ‘cure’ has generally been replaced by ‘remission’, which is usually defined as <5 µg/dL (<138 nmol/L) or urinary free cortisol <28-56 nmol/day (<10-20 µg/day) within 7 days of selective tumour resection (20). According to these criteria, remission rates of 70-98% have been reported (8, 14, 21, 22), but also remission rates of 100% and 69% were reported in two large paediatric CD series which used stricter definitions of remission, namely post-TSS serum cortisol of <1 µg/dL (28 nmol/L) and <1.8 µg/dL (50 nmol/L), respectively (7, 23). In summary, remission rates of ~70% or greater are now expected from specialist centres with experience of paediatric CD. The most common complication of TSS was post-operative diabetes insipidus, present in 5% of patients at discharge from neurosurgical care (8). Overall, TSS is effective and safe first-line treatment for paediatric CD. An endo-nasal approach with endoscopy may be also used for access to the pituitary and this is now gaining popularity, particularly in adult patients. A small paediatric series of patients from an English reference centre showed biochemical remission in 5 out of 6 patients, with no recurrence after a mean follow-up of 55 months (24). Second-line therapy in the form of reoperation, pituitary radiotherapy, bilateral adrenalectomy or long-term medical therapy to control cortisol synthesis may be required in approximately 30% of cases.

## Recovery of the hypothalamic-pituitary-adrenal axis

Adrenal insufficiency follows successful pituitary adenoma resection and may persists for many months. A median time of recovery of the HPA axis in 106 paediatric patients successfully treated by TSS at the NIH was 12.3 months with a range of 3-35 months (25). The level of pre-operative urinary free cortisol related to recovery time with high values being associated with longer intervals of recovery of normal adrenal function.

## Recurrence of Cushing’s disease following remission

**Figure 1:**
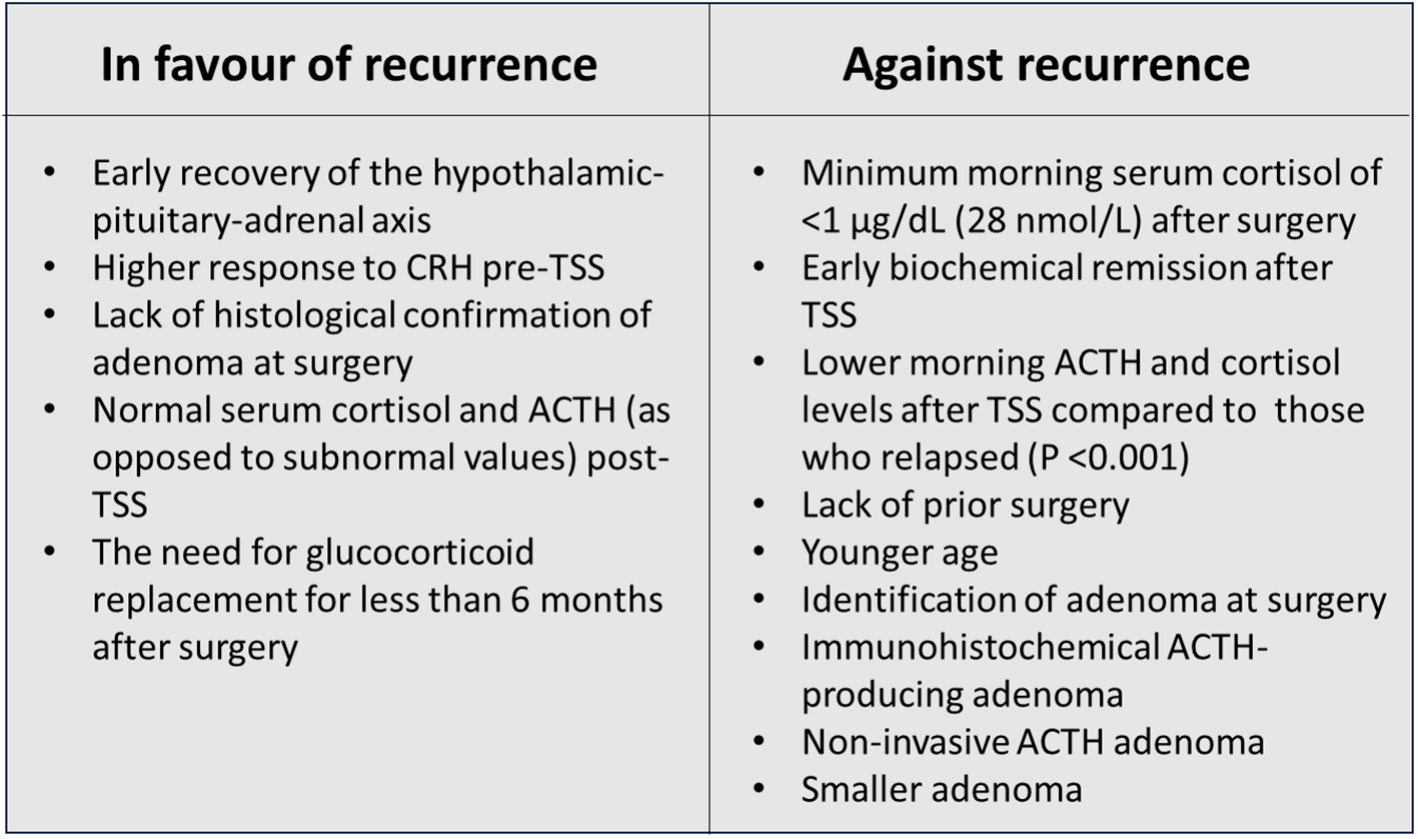
Factors in favour and against recurrence of paediatric Cushing’s disease.

There are relatively few studies of recurrence of CD following treatment during the paediatric age range. Yordanova et al. studied long-term follow-up of 21 paediatric CD patients in remission following definitive TSS or pituitary radiotherapy. During an interval of 2 to 7.6 years, the recurrence rate was 14.3% (26). The most comprehensive data comes from the NIH. Interesting results were reported showing a relationship (p=0.0342) between a shorter HPA axis recovery time and the likelihood of recurrence of CD (25). All patients with recurrence of hypercortisolism had recovery of the HPA axis by 15 months post-TSS. It is helpful to look at risk of recurrence in the context of prediction of short-term remission following TSS – because the two phenomena are linked. Essentially the factors significantly predicting remission (P=<0.05) after TSS are: lack of prior surgery, younger age, identification of adenoma during surgery, the presence of a positive ACTH-producing immunohistochemical adenoma and a non-invasive and smaller adenoma (8) (**Figure 1**). All these factors favour complete removal of the adenoma. In a series of 78 paediatric CD patients from the NIH, 94% had sustained remission for 5.8 – 18.3 years with 6 patients showing recurrence of CD. Children who remained in remission had; lower morning ACTH and cortisol levels during the post-operative period after TSS compared to those who relapsed (P <0.001). Relapse was associated with; higher cortisol response to CRH pre-TSS, lack of histological confirmation of adenoma at surgery, normal serum cortisol and ACTH (as opposed to subnormal values) post-TSS and the need for glucocorticoid replacement for less than 6 months after surgery, ie a rapid recovery of the HPA axis (10). The confirmation of these factors, in an expanded report of 179 NIH patients (8) reinforced the message that a positive predictive value for lasting remission (96%), was associated with a minimum morning cortisol of <1 µg/dL (28 nmol/L) during the immediate post-operative period. Paediatric patients harbouring somatic *USP8* mutations were reported to have a higher likelihood of recurrence of CD following TSS compared with patients without mutations (46.2% vs 10.3%, P=0.009) (27).

## Paediatric radiotherapy

External pituitary radiotherapy (RT) is an optional second-line therapy for patients not in remission after TSS. However, this treatment in children is controversial because of concern related to cognitive effects and possible post-RT hypopituitarism. Corticotroph microadenomas respond well to conventional fractionated external RT, as first documented in 1977 (28). An English series of 7 paediatric patients reported treatment by a 6-MV linear accelerator delivering a dose of 45 Gy in 25 fractions over 35 days, which induced remission in all subjects at a mean interval of 0.94 years (range 0.25-2.86) (29). These data were later confirmed in a multi-centre study focusing on the gamma knife radiotherapy technique on 24 children with recurring CD, which induced remission in 87.5% of subjects after a mean interval of 12 months (30). The effects of RT take several months during which control of hypercortisolism is required using medical therapy.

## Bilateral adrenalectomy

Bilateral adrenalectomy may represent a life-saving procedure in children with very severe hypercortisolism and life-threatening clinical morbidities *per se* or which may prevent safely approaching more definitive treatments, as TSS. Virtually, bilateral adrenalectomy has a remission rate of 100%, as it removes the source of cortisol production, and only patients with subtotal or incomplete surgical removal may experience recurrence after bilateral adrenalectomy (4). However, bilateral adrenalectomy requires life-long replacement treatment with glucocorticoids and mineralocorticoids and may be associated with Nelson’s syndrome. The current definition of Nelson’s syndrome is ‘radiological progression or new detection of a pituitary tumour on thin-section MRI’ (31). Clinically this is associated with increasing levels of ACTH causing hyperpigmentation, that may even occur several years after bilateral adrenalectomy (31). Although few paediatric series have been reported, a mean interval of 8.4 years post-adrenalectomy was reported in one series (32). The mean cumulative incidence of Nelson’s syndrome was considerably higher (45%, 25–67%) compared to results in adult patients (31). This emphasizes the importance of life-long follow-up of all paediatric CD patients irrespective of the therapy they have received.

## Medical therapy

Whereas extensively used in adult CD patients (33), few reports are available for the medical treatment of children with CD. Ketoconazole, a multi-enzyme steroidogenesis inhibitor, is the most frequently reported drug in children with CD, mainly in patients needing fast symptomatic relief from CS comorbidities and in patients waiting for pituitary radiotherapy to be effective (4). A French study reported the effects of “low-dose” mitotane, an adrenolytic drug used in adrenocortical cancer and in patients with very severe CS. In 9 CD children, there was significant improvement in weight and growth rate after 12 months of treatment, as well as a general improvement of clinical features, although the high rate of reported adverse events suggests caution in the widespread use of this treatment in children (34). Therapeutic trials are currently in progress in children using the 11-beta hydroxylase blocking agent osilodrostat, which is now approved for treatment of adults with CD (35).

## Linear growth and pituitary function following TSS and/or radiotherapy

**Figure 2:**
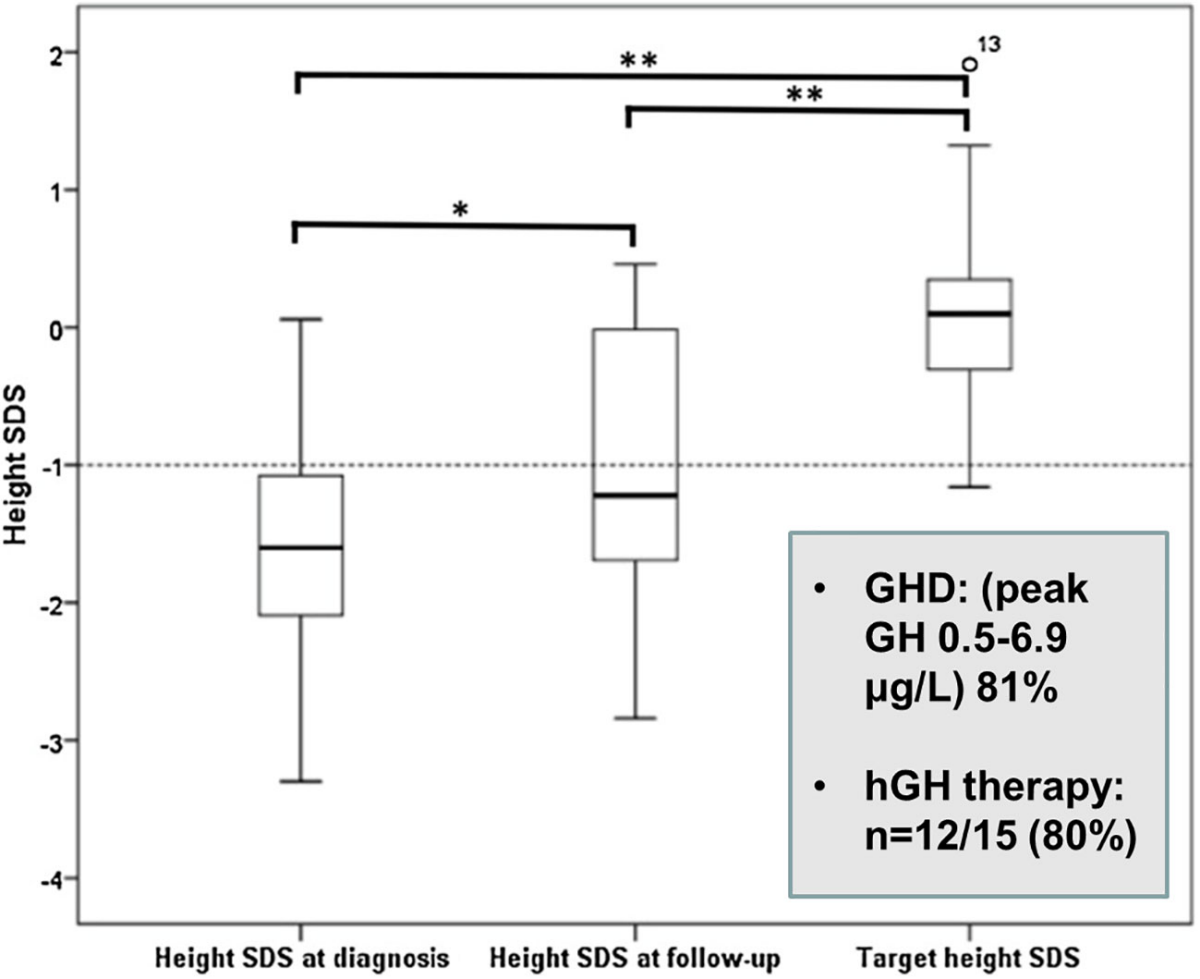
Height SDS values at diagnosis, latest assessment and target height in paediatric Cushing’s disease (26). *p = 0.033, **p = 0.000.

Growth hormone deficiency (GHD) is common after TSS in children (5) and may also be a complication of pituitary RT (9, 20). In an English series, GHD occurred in 86% of paediatric patients treated with RT, but after 10 years of follow-up 3/4 boys showed recovery of GH secretion to a peak GH value of >10 ng/ml (9). Gonadotrophin deficiency was also present in 9/20 subjects causing delayed puberty and several required sex steroid replacement (26). A major goal in the management of paediatric CD is the restoration of normal linear growth during remission after successful TSS. As CD often presents shortly before or during puberty, the time for catch-up growth is frequently limited and decreased adult height in paediatric CD has been well documented (5, 11, 36) (**Figure 2**). We advocate testing for GH deficiency 3 months after TSS and a low threshold for hGH replacement, if necessary in combination with a GnRH analogue (4).

There are follow-up data on two other variables, namely body composition and bone mineral density. In 14 patients treated by TSS alone (n=8) or TSS followed by external pituitary radiotherapy (n=6), body mass index (BMI) SDS was elevated at +2.7 (0.8 - 5.1) at diagnosis. At a mean interval of 4.1 (1.1 -10.7) years after remission of hypercortisolaemia (postoperative serum cortisol <50 nmol/L), BMI SDS remained elevated above the mean at 1.7 SDS, being lower than at diagnosis (P < 0.05), but elevated compared to the normal population (P < 0.01) (37). It is often difficult for children to normalise their BMI after remission. Careful dieting is required to minimise the risk of continuing insulin resistance.

Bone mineral density (BMD) is frequently reduced at diagnosis of paediatric CD (4). Two groups of patients were studied using DEXA, the first comprising 8 patients, mean age 12.4 yr (8.2 – 16.8 yr) had a mean L2-L4 volumetric BMD at diagnosis of -1.04 (-3.2 – 0.11) with values corelating negatively with midnight serum cortisol (P = < 0.05) (38). The second group comprised 11 subjects with hypercortisolaemia in remission following TSS or external RT, studied at a mean of 4.5 yr after remission, during which 8/11 had received hGH replacement (37). Mean L2-L4vBMD SDS was -0.38 (-1.0 – 0.13) with mean femoral neck areal BMD SDS of 0.14 (-1.62 – 2.46). These data show variable BMD SDS at diagnosis and near normal BMD SDS after induction of remission in paediatric CD (38).

## Conclusions

Paediatric CD can be regarded as a niche disorder, which ideally requires joint input from paediatric and adult endocrinologists in terms of diagnosis and therapeutic strategy. Ideally a small number of tertiary centres should be reserved for the management of these patients. Following diagnosis, it is of crucial importance that a pituitary surgeon with prior experience of transsphenoidal surgery in children is identified for referral and becomes part of the management team. Complete selective excision of the corticotroph adenoma is difficult due to its very small size, but is directly related to long-term post-operative remission and quality of life. In experienced hands, the prognosis is good and the rate of recurrence is low. Post-operative challenges relate to catch-up linear growth and resumption of normal body composition. Life-long follow-up is required.

## Author contributions

MS: Conceptualization, Writing – original draft, Writing – review & editing. RF: Conceptualization, Formal analysis, Writing – review & editing.
